# Microbial Endocrinology in the Microbiome-Gut-Brain Axis: How Bacterial Production and Utilization of Neurochemicals Influence Behavior

**DOI:** 10.1371/journal.ppat.1003726

**Published:** 2013-11-14

**Authors:** Mark Lyte

**Affiliations:** Department of Immunotherapeutics and Biotechnology, School of Pharmacy, Texas Tech University Health Sciences Center, Abilene, Texas, United States of America; University of North Carolina at Chapel Hill School of Medicine, United States of America

## Introduction

The ability of bacterial pathogens to influence behavior has been recognized for decades, most notably bacteria that directly invade the nervous system. However, increasing evidence is mounting that microorganisms may directly interact with elements of the host's neurophysiological system in a noninvasive manner that ultimately results in modification of host behavior. This ability of microorganisms contained within the microbiome to influence behavior through a noninfectious and possibly non-immune-mediated route may be due to their ability to produce and recognize neurochemicals that are exactly analogous in structure to those produced by the host nervous system. This form of interkingdom signaling, which is based on bidirectional neurochemical interactions between the host's neurophysiological system and the microbiome, was introduced two decades ago and has been termed microbial endocrinology [1]. Many of the neuroendocrine hormone biosynthetic pathways that are more commonly associated with eukaryotic cells are found in prokaryotic cells, and the acquisition of such neurochemical-based synthesis pathways by eukaryotic systems is believed to be due to lateral gene transfer from bacteria. Approaching the microbiome from a microbial endocrinology-based vantage point may provide an understanding of the specific pathways by which microorganisms may influence behavior and thereby lead to new approaches to the treatment of specific mental illness based on modulation of the microbiome-gut-brain axis.

## What Is Known—Associations of Microbes with Behavior

The ability of pathogens to influence host behavior has been well recognized for decades. Among the most dramatic examples is *Toxoplasma gondii* infection of rodents that results in such a profound decrease in anxiety-like behavior that infected animals no longer show fear of feline predators [2]. In humans, individuals suffering from inflammatory bowel diseases, which are characterized by altered microbial diversity, have demonstrated poorer emotional functions such as anxiety and depression [3]. What many of these studies, which demonstrate the ability of a specific pathogen or altered microbiome to influence host behavior, have in common is that all produce immune-related sequelae that result in the release of host immune factors, such as cytokines and inflammatory mediators, that have known neuronal targets both within the central nervous system (CNS) and the enteric nervous system (ENS) [4].

While the sequence of pathogen infection resulting in immune activation that then ultimately results in an alteration of behavior is well recognized, it is perhaps somewhat surprising to learn that increasingly other studies are reporting the direct, *non-immune*, *non-infectious*, related ability of microbes to influence behavior. The first study that demonstrated the ability of a bacterium within the gut to influence behavior was shown in a series of studies utilizing *Campylobacter jejuni* in mice [5]. In this series of studies, a low per oral dose of *C. jejuni* was able to induce anxiety-like behavior in mice through a vagal-mediated pathway in the absence of any immune activation [6]. Since these early reports in the 1990s, this microbial-gut-brain axis has been the subject of growing investigation and has even engendered the use of the term “mind-altering bugs” [7]. While most studies of the microbial-gut-brain axis have centered on the ability of certain bacteria, whether commensal, pathogen, or probiotic, to effect a plethora of neural substrates both within the CNS and ENS, less attention has been centered on properties of the microbes themselves, which from an evolutionary standpoint strongly suggest that the microbiome is in constant communication with the host's neurophysiological system [8]. As will be discussed, the ability of bacteria to synthesize and recognize the very same neurotransmitters that are found in the vertebrate host suggests a bidirectional environment where the microbiome can influence the host and the host influence the microbiome. This level of communication between host and microbiome and its mediation by a commonly shared evolutionary pathway of intercellular signaling suggest that “they monitor us” and “we monitor them” [8]. (*It should be noted that the viral component of the microbiome, or virome, is not addressed in this review.)*

## Microbiology Endocrinology—Intersection of Host Neurophysiology and Microbes

The extent of the prevalence of neuroendocrine hormones in nature that are exactly the same in structure, and most interestingly biochemical synthesis pathways, is often not fully appreciated. For example, the neuroendocrine hormone norepinephrine is found in plants, as well as in insects and fish, and most critically from the standpoint of microbiologists, in microbes [9]. Indeed, due to these same shared biochemical pathways, the existence of neurochemical-based cell-to-cell signaling pathways such as those in humans has been proposed to be due to late horizontal gene transfer from bacteria [10].

The role of neuroendocrine hormones, especially those biogenic amines related to the stress response, in the pathogenesis of infectious disease has become increasingly recognized following the first reports in the early 1990s that documented the ability of catecholamines to directly stimulate bacterial growth and alter virulence factor production [11]. These initial studies led to the proposal of the field of microbial endocrinology, which in effect represents the intersection of host neurophysiology with the microbiome in which neuroendocrine-bacterial interactions are a governing mechanism (for review, see [12]). More recently, investigators have shown that the neuroendocrine outflow from a host neurophysiology event such as stress-mediated release of flight-or-flight hormones can alter gene expression in a number of pathogens as well as conjugative transfer between enteric bacteria [13]. It should also be noted that while this review centers on monoamine-based compounds such as the catecholamines, other neuroactive compounds not typically thought to be associated with the microbiome can be produced in great enough concentration to affect the pathogenesis of host disease. For example, synthesis of benzodiazepine receptor ligands by gut bacteria can contribute to the development of encephalopathy that can accompany fulminant hepatic failure by accumulating in the brain and enhancing GABAergic transmission [14].

## Moving beyond Infection to Host Behavior

The ability of bacteria to produce neuroendocrine hormones suggests that the interaction of the microbiome with the host may go far beyond the role of such host neuroendocrine-bacterial interactions in infectious disease. It is perhaps underappreciated by most microbiologists that the gut is a highly innervated organ that possesses its own nervous system known as the enteric nervous system (ENS) that is in constant communication with the central nervous system (CNS) through nerves such as the vagus, which directly connect portions of the gut to the brain (for a review of gut neuroanatomy, see [15]). Further contributing to the amount of information obtained in the gut are the luminal epithelial chemosensors, which can respond to and transmit information regarding bacterial metabolites such as neuroactive compounds that are contained within the luminal space [16]. This gut-to-brain communication has been the subject of intensive study for many years and is recognized to play an important role in the ability of gut-related pathologies to also result in mental health–related issues such as depression [17].

The microbiome produces a wealth of neuroactive substances such as catecholamines, histamine, and other compounds that can stimulate host neurophysiology either through direct interaction with receptors within the gastrointestinal tract or following absorption/passive diffusion through the gut wall and entering into the portal circulation. Within the gut, neuronal projections from the ENS can innervate the entire length of the microvilli [15], [18]. Coupled with the presence of a myriad of cells within the gastrointestinal tract whose function encompasses the gathering of information regarding the composition of the luminal environment, such as enterochromaffin cells and luminal epithelial chemosensors [16], there is undoubtedly a wealth of sensory information that is being detected by the host emanating from the gut lumen that has the potential to be interpreted and acted upon by components of the CNS such as the brain. Probably one of the most dramatic examples of the interconnectedness of the gut microbiome with the host neurophysiological system is the report by Neufeld *et al.*, which demonstrated that excitability of gut sensory neurons located within the myenteric plexus of the ENS (isolated from jejunal segments of the intestine) relied on the presence of the normal commensal microbiota for proper functioning [19].

As such, the number of reports demonstrating that bacteria within the gut can be detected by the brain with consequent changes in behavior has been increasing following the initial report utilizing *C. jejuni* [5] as described above and subsequent reports identifying the neural substrates both within the brain and vagal-mediated gut-to-brain pathway [6]. For example, the ability of certain probiotic bacteria, such as *L. rhamnosus* (JB-1), to influence emotional behavior in mice has been shown to be mediated via GABA receptors [20]. Changes in diet such as feeding of meat, which can dramatically alter the composition of the microbiome, have also been shown to improve memory and learning in rodents [21]. It should not be surprising then to learn that the intestinal microbiome plays a critical role in the development of even the brain itself from the time of birth [22].

## Microbial Endocrinology as a Central and Unifying Mechanism

The identification of mechanisms by which the microbiome influences host neurophysiology and ultimately behavior will be crucial to understanding how the composition of the microbiome influences behavior. It is therefore proposed that approaching this question through the use of microbial endocrinology will provide one (of many possible) route(s). Recognition of the neuroendocrine-hormone-producing capacity of microbes will hopefully spur new investigation into the ability of members of the microbiome to produce neuroactive compounds that have specific targets within the host neurophysiological system.

There are a number of substantial methodological issues that will need to be addressed. The innervation of the gut is not homogenous throughout its length [15]. Neither is the microbiome. As such, it will be crucial to understand how one microbial species that may produce a neuroactive compound may have a behavioral effect while in one part of the gut and not another. Sampling of the microbiome adjacent to specific areas of innervation within the gastrointestinal tract and associating it with the route via which sensory information obtained within the ENS is communicated to the CNS by the intrinsic primary afferent (sensory) neurons that follow vagal or spinal afferent routes may be one approach. Another substantial methodological issue yet to be fully addressed is that the capacity of microbes to produce neuroactive components is dependent on the availability of suitable substrates, just as it is for mammalian cells. The role of diet therefore must also be a consideration in evaluating the capacity of the microbiome to produce neuroactive compounds. This also applies to any *in vitro* studies of a specific species' ability to produce neuroactive compounds as most microbiological media is not reflective of what would be present *in vivo*.

We are just at the beginning of comprehending the meaning of gut-to-brain microbiome interactions and what it ultimately means for host homeostasis including behavior. Recently, the role of bacteria in determining appetite and food preference was proposed [23], [24]. It is intriguing to speculate that microbes play a far larger role in normal homeostasis than previously imagined.
